# Characterization of Actinomycetes and Trichoderma spp. for cellulase production utilizing crude substrates by response surface methodology

**DOI:** 10.1186/2193-1801-3-622

**Published:** 2014-10-21

**Authors:** Tanveer Pirzadah, Shashank Garg, Joginder Singh, Ashish Vyas, Manish Kumar, Naseem Gaur, Madhu Bala, Reiaz Rehman, Ajit Varma, Vivek Kumar, Manoj Kumar

**Affiliations:** Department of Bioresources, University of Kashmir, Srinagar, 190006 Jammu & Kashmir India; Department of Microbiology, School of Biotechnology and Biosciences Lovely Professional University, Punjab, India; AIRF, Jawaharlal Nehru University, New Delhi 67, India; International Centre for Genetic Engineering and Biotechnology (ICGEB), New Delhi 67, India; Institute of Nuclear Medicine and Allied Sciences (INMAS), DRDO, New Delhi 54, India; Amity Institute of Microbial Technology, Amity University Uttar Pradesh, Noida, 201303 India

**Keywords:** Response surface methodology, CMC, Wheat bran, Avicel, Zymogram, Circular dichroism

## Abstract

Laboratory bench scaling was done and an average of 1.85 fold increase by Response Surface Methodology (RSM) optimization was obtained. It was found that the predicted value (4.96 IU/ml) obtained by RSM is in close accordance with observed activity 5.14 IU/ml. Endoglucanases are mainly induced by CMC while Wheat bran (natural substrate) exoglucanase is more active when induced by avicel and cellulose. Addition of substrate beyond a level caused inhibition of cellulase production. The molecular weight of protein as determined by SDS-PAGE is very similar to molecular weight of cellulase of *Trichoderma viride* (*T. viride*) cellulase and *Trichoderma reesei* (*T. reesei*) endoglucanase. *T. reesei* β-glucosidase has high enzymatic activity on CMC substrate when compared with *T. viride* β-glucosidase. Secondary structure analysed by using Circular Dichroism confirmed that composition of celluase system is very similar to other analysed species. The cellulase was found to be active in pH range of 4.8-5.5; while temperature range varied from 50°C to 70°C. Although the enzymatic activity produced by mutants were lesser than the parent, but in one case mutants of *Trichoderma* reesei’s BGL has shown higher activity on cellulose.

## Background

Microbial cellulase have wide spread application in textile, pulp, brewing, food and agriculture industry. Because of its commercial importance and complexity of structure, research of many labs and industries are revolving around cellulase producing microbes, its production and its characterization. Structurally, it has two domains, cellulose binding module (CBM) and a catalytic domain (CD). Cellulase family contain three groups of enzymes, endo-(1,4)-β-D-glucanase (EG), exo-(1,4)-β-D-glucanase (CBH) and β-glucosidase (BGL). EG breaks o-glycosidic bonds which are present internally but randomly. CBH attacks cellulose chains at the ends, to release β-cellulobiose. BGL acts on β-cellulobiose and generates glucose (Kuhad et al. 2011). They act together to form glucose from cellulose (Sukumaran et al. 2005). Characterization of cellulase is challenging task for enzymologists because it’s a complex of multiple subunits, and mode of action and requirements are different from one another.

There are many microbes, including bacteria and fungi that produce cellulase. *Cellulomonas, Pseudomonads, Bacilli* and *Actinomycetes* species of bacteria; *Aspergillus*, *Humicola*, *Trichoderma* and *Penicillium* species of fungi have got primary importance in research and industrial purpose because of their selective advantage over other species. *H. insolens*, *Thermomono sporafusca*, *A. niger*, *T. reesei* and few other species are exploited for industrial purpose. Complexity of fungal cellulase is less than bacterial cellulose and former is produced extracellularly in large quantity (Sukumaran et al. 2005). *Trichoderma* genus is found to produces cellulase enzymes with high enzymatic activity as compared with other genera (Miettinen-Oinone et al. 2002).

Characterization of cellulase for structure and activity is required to optimize the best combination of physical and chemical parameters including temperature and pH optimum, mode of action on different substrates, nutrient requirement and mode of culture (Beldman et al. 1985). Statistical tools of Design of Experiments are latest trends in bioprocess developments and process optimization. Response surface methodology (RSM), developed by Box and his collaborators, is a collection of mathematical and statistical techniques for designing experiments, building models, searching optimum conditions of factors for desirable responses, and evaluating the relative significance of several affecting factors in the presence of complex interactions (Bezerra et al. 2008). RSM is used to determine the range of controllable variables and optimal production conditions, followed by generation of model polynomial equation of the bioprocess, with estimations of the relationships between controllable variables and observed results. RSM has been used for modeling and optimization of process conditions, such as enzyme-catalyzed reaction conditions, adsorbents-aided dye removal from waste waters and production of enzymes like lipase, glucosidase, and extracellular polysaccharides (Zambare et al. 2011).

## Materials and methods

### Procurement of *Trorichoderma*strains

*Trichoderma reesei* (*T. reesei*) (MTCC No. 3114) and *Trichoderma viride* (*T. viride*) (MTCC No. 164) were procured from IMTECH, Chandigarh, India and revived on Malt extract agar. *T. reesei* and *T. viride* were grown at 28°C for 7 days and 5 days respectively. While 3 mutant lines of *Trichoderma* species i.e. *T. reesei* (ITCC No. 4026), *T. viride* (ITCC No. 6585) and *T. Koningii* (ITCC No. 5201) were collected from ITCC New Delhi. The cultures were preserved at 4°C on PDA slants.

### Soil microbes

The soil samples were collected from high altitude of Leh, Jammu & Kashmir, India. Actinomyctes specific medium (Actinomycetes Isolation Agar from HiMedia) was used to grow specific culture using serial dilution approach (10^−1^ to 10^−5^) of the rhizospheric soil from Sea-buckthorn (*Hipopphae rhamnoides*) populated area. Followed by inoculation on same media containing plates at 28°C for 3-4 days. Standard protocol by Baker and O'Keefe was followed with slight modification (Baker et al. 1984).

#### Inoculum preparation and screening for cellulase production

For inoculum preparation, standard protocol by Reddy et al. was followed and 50 ml of the media with 2 ml spore inoculum was incubated in shaker incubator at 28°C (Reddy et al. 1998). *Trichoderma* and actinomycetes cultures were inoculated in potato dextrose medium and actinomycetes isolation medium respectively with 0.5% congo red dye for determining cellulase production.

#### Cellulase production

Fungal cultures were grown in 500 ml flask containing Mendel and Reese medium (Mandels et al. 1974). Constituents were mixed and made to 250 ml with sterile distilled water, followed by addition of specific substrates or carbon sources [viz. 10 g/l of CMC, cellulose, RC, RC-CMC, WB respectively]. Culture was incubated at 28°C at 100 RPM. 10 ml samples were withdrawn on 8^th^ day of incubation and mycelia was separated by centrifugation at 1000 RPM for 20 min. Supernatant obtained was used as source of crude enzyme (Vyas et al. 2005).

#### Partial purification

Crude enzyme was precipitated by using Ammonium Sulphate and then dissolved in 10 ml of 50 mM of sodium acetate buffer (pH-5.5). This partially purified enzyme was dialyzed against 30 mM sodium acetate buffer (pH-5.5) at 4°C and same enzyme was used for activity assays.

#### Qualitative enzyme assays

Double-layer plate assay, as described by Mateos et al., was used for qualitative assaying of enzyme preparation (Mateos et al. 1992). Bottom layer of 0.7% agarose and top layer with 0.2% agarose were used for the assay. Cellulolytic activities were revealed by flooding the plates with an aqueous solution of 1% Congo red for 30 min followed by decolourilazion by 1M NaCl. Interaction of congo red with contiguous polysaccharides and hemicellulosic galactoglucomannans provides the basis of assays for β-glucanase (Teather et al. 1982). Mendel and Reese medium was prepared with specific components and agar. Plates were flooded with the Congo red solution. After 1 hour, discoloration was done with 1 M NaCl.

#### Quantitative enzyme assays

Glucose standard curve was plotted between glucose concentration and OD at 540 nm to draw standard curve for calorimetric analysis to quantify enzyme activity.

### Assay of endoglucanase activity (CMCase assay)

Endoglucanase activity was measured as per method described by Mandels et al. (1976) with slight modification. Reaction mixture consisting of 0.5 ml of 1% CMC in citrate buffer at pH 4.8. The reaction was stopped by adding 3 ml of DNS in the boiling water. After cooling down, the absorbance was taken at 540 nm (Ghose 1987). 1 unit (IU) of endoglucanase activity is defined as the amount of enzyme which liberated 1 μmole of glucose/min under standard conditions.

### Assay of FPase

FPase activity was assayed by measurement of reducing sugars in the reaction mixture containing Whatman No. 1 filter paper (1.0 cm × 6.0 cm ~50.0 mg) as substrate in 1ml sodium citrate buffer (50 mM, pH 4.8) at 50°C for 60 min (Mandels et al. 1976). 1 unit (IU) of FPase activity is defined as the amount of enzyme which liberated 1μmole of glucose/min under standard conditions.

### Assay of BGL activity

For β–glucosidase activity, 0.5 ml of 1% salicin, prepared in citrate buffer (pH 5.5) was used as substrate for enzyme activity (Ahmed et al. 2009). 1 IU of BGL is defined as amount of enzyme which liberates 1 micromole of glucose from salicin per min under assay conditions.

#### Screening of significant factors components by Plackett-Burman design

Screening of the significant factors for the media optimization was done by Plackett-Burman experimental design (Rajendran et al. 2007). Experiments were done for evaluating significant factor such as CMC, WB, RC, peptone, pH, MgSO_4_.7H_2_O and KH_2_PO_4_. 12 runs of experiment were carried out to evaluate these parameters for cellulase activity as response. The analysis of response was done with the presentation of Pareto chart.

#### RSM (Response Surface Methodology) for media optimization

Significant factors revealed by PB design were optimized for cellulase production. Central Composite Design (CCD) in statistical software package “Design Expert 8.0.7.1” was used to obtain a quadratic model, consisting of factorial trials and star points to estimate quadratic effects and pure process variability with cellulase production as response (Table 1) (Garai et al. 2012).

**Table 1:** **Ranges of variable used for CCD**

Coded level					
Variable	−2	−1	0	1	2
CMC (g/l)	8	10	12	14	16
KH_2_PO_4_ (g/l)	1	2	3	4	5
Peptone (g/l)	0.5	1.5	2.5	3.5	4.5
pH	3	4	5	6	7

#### SDS PAGE and zymography

Sodium Dodecyl Sulphate Polyacrylamide Gel Electrophoresis (SDS-PAGE) was performed on 5% stacking gel and a 15% separating gel, according to the method described by (Laemmli 1970) to determine the molecular weight of crude and partially purified cellulase (Laemmli et al. 1970). To find cellulolytic activity of cellulase, Zymography technique was used (D'Avila-levy et al. 2012; Schwarz et al. 1987). CMC (0.2%) was added to SDS PAGE before polymerization for *T. reesei* and *T. viride* to observe the activity cellulases on the gel*.*

#### Circular dichroism for secondary structure analysis

Cellulase samples (MW 48 KDa) of *Trichoderma reesei, Trichoderma viride,* were subjected for structural characterization through circular dichroism (CD). The isothermal studies of cellulase by CD measurements were carried out with Chirascan (Applied Photophysics equipped with a QUANTUM N.O.R.T.H.W.E.S.T.-TC125). The instrument was calibrated with d-10-camphorsulfonic acid (Schmid et al. 1997). All the isothermal CD measurements were recorded at 25°C. Spectra were collected at 20 nm/min scan speed with 0.1nm data pitch and response time of 2 s. Each spectrum was the average of 10 scans. The Far-UV CD spectra (200–260 nm) were taken at protein concentrations of 0.02 mg/ml in a cell of 0.1 cm path length. All spectra were smoothed by the Savitzky-Golay method with 25 convolution width.

Ellipticity values (θ) in mdeg, obtained from the instrument readings, were expressed in terms of mean residue ellipticity [θ] using the following equation:


Where *M* is the mean residue mass, l the path length (0.1 cm), C is the concentration in g/ml and [θ] is the mean residue ellipticity (Wallace et al. 2002). CD data was further processed using CDNN software for the prediction of secondary structure.

## Results and discussion

### Isolation and identification of Actinomycetes

Novel isolate from the soil sample collected from high altitude of Leh, Jammu & Kashmir, India was identified as actinomycetes by Gram staining and Methylene blue tests.

### Screening of the *Trichoderma*and Actinomycetes strains for cellulase production

All the organisms under study which were incubated with congo red, produced cellulases as observed due to change in color of medium. This served as joint screening test for production of cellulases.

### Congo red (CR) test for enzyme production

Clear zone of degradation of substrate in double layer plate assay reveals the production of enzyme by organisms under study. Although all fungi and actinomycetes produced the zone of clearance, *T. reesei* produced the largest zone.

Qadri et al. (2013) have also shown an important step towards the fungal characterization collected from the Western Himalayas and optimized the isolation and screening approach for industrial scaling.

### Quantitative analysis enzyme assay on different substrates

#### CMCase assay

**Figure 1:**
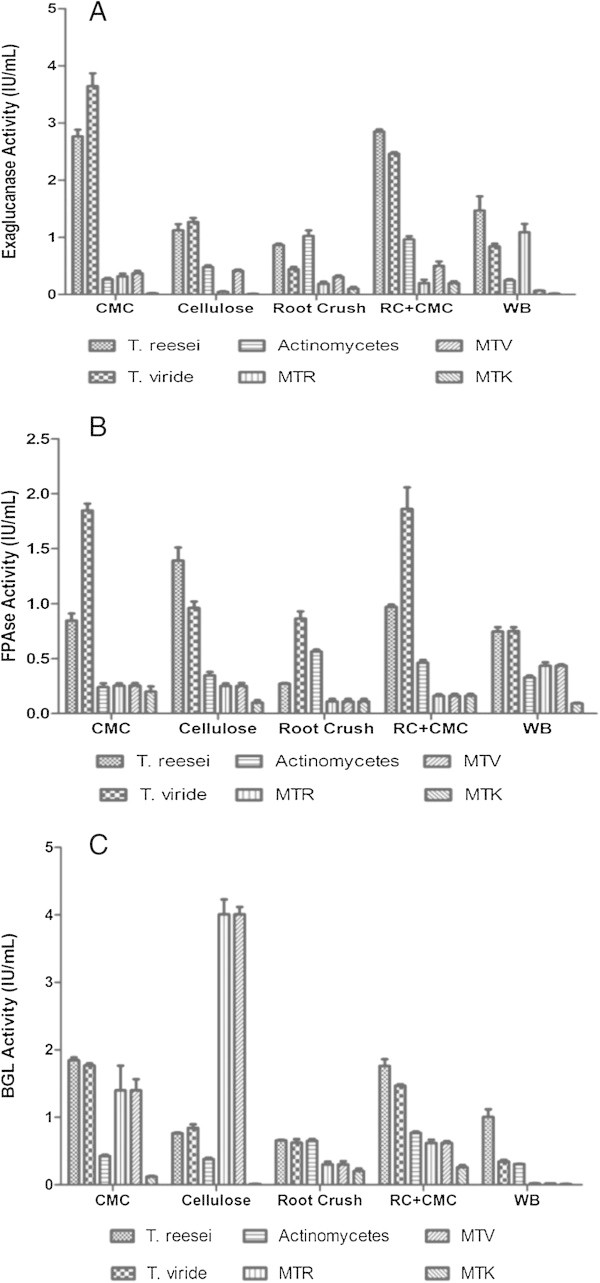
**Activities of different cellulases from respective organisms over different substrates. (A)** Exaglucanase activity of the 6 organisms under study cultured in different carbon sources viz. CMC, Cellulose, Root Crush, Root Crush + CMC and Wheat Bran. **(B)** FPAse activity of the 6 organisms under study cultured in different carbon sources viz. CMC, Cellulose, Root Crush, Root Crush + CMC and Wheat Bran. **(C)** β-glucosidase activity of the 6 organisms under study cultured in different carbon sources viz. CMC, Cellulose, Root Crush, Root Crush + CMC and Wheat Bran.

Endoglucanase activity of crude cellulase produced by the strains on the 8^th^ day of fermentation was measured by CMCase assay (Figure 1A). *T. viride* grown on CMC exhibited highest activity (3.646 IU/ml), followed by *T. reesei* on RC + CMC (2.849 IU/ml). Actinomycetes grown on RC have shown good activity (1.024 IU/ml) when compared with other substrates. Mutants MTK, MTR, MTV produced less activity than parent strains.

#### FPA assay

*T. viride* (Figure 1B) grown on RC + CMC revealed highest activity than remaining substrates and slightly less activity on CMC i.e. 1.846 IU/ml. Actinomycetes (Figure 1B), grown on RC has shown maximum activity (0.564 IU/ml) when compared with remaining substrates. Least activity was observed for MTK grown on wheat bran (Figure 1B).

#### BGL assay

Figure 1C depicts the BGL activity of all strains of the crude cellulase obtained on the 4^th^ day of fermentation. Mutants of *T. reesei* grown on cellulose exhibited highest activity when compared with other species (4.01 IU/ml) and least activity observed on wheat bran by MTK (0.26 IU/ml). Actinomycetes grown on RC-CMC (0.768 IU/ml) produced good activity when compared with other sources.

Similar data analysis carried out by different researchers (Beldman et al. 1985; Haq et al. 2005 & Vyas et al. 2005) has been effectively scaled with wider range of crude enzymes obtained from efficient strains and the fast fermentation approach adapted in the current research work.

### Screening of factors by Plackett-Burman experimental run

#### Screening of the most significant factor components by Plackett-Burman design

All runs were done in duplicates and the mean value of activity was noted as response. The highest value of 3.6 IU/ml was obtained in the 8^th^ run, while lowest value was recorded as 0.26 IU/ml in the 1st run. The data shows a large variation between lowest and highest activity which supports the actual coding and quantity of medium components we had assigned (Table 2).

**Table 2:** **Experimental runs for Plackett – Burman Design**

Run	A CMC	B WB	C RC	D Peptone	E pH	F MgSO _4_	G KH _2_PO _4_	H*	J*	K*	L*	Response (Activity(IU/ml))	
												Predicted	Actual
1	−1	−1	1	−1	1	1	−1	1	1	1	−1	0.26	0.18
2	1	1	1	−1	−1	−1	1	−1	1	1	−1	0.80	0.88
3	1	−1	1	1	−1	1	1	1	−1	−1	−1	2.40	2.58
4	−1	−1	−1	−1	−1	−1	−1	−1	−1	−1	−1	0.40	0.32
5	1	1	−1	1	1	1	−1	−1	−1	1	−1	0.90	0.94
6	1	1	−1	−1	−1	1	−1	1	1	−1	1	0.60	0.54
7	1	−1	−1	−1	1	−1	1	1	−1	1	1	3.40	3.98
8	1	−1	1	1	1	−1	−1	−1	1	−1	1	3.60	3.2
9	−1	1	−1	1	1	−1	1	1	1	−1	−1	1.40	1.11
10	−1	−1	−1	1	−1	1	1	−1	1	1	1	0.94	0.87
11	−1	1	1	1	−1	−1	−1	1	−1	1	1	0.40	0.38
12	−1	1	1	−1	1	1	1	−1	−1	−1	1	1.30	1.6

*Dummy variables.

Plackett-Burman design (PBD) is the most recommended screening design and mathematical approach used in mathematical applications in engineering because if its ability to estimate all main effects with the same precision (Antony 2008).

#### Elimination of insignificant factor by data analysis

Generally, a large *t-*value associated with a low *P-*value of a variable indicates a high significance of the corresponding model term. CMC, peptone, K_2_HPO_4_, and MgSO_4_ displayed a positive effect for enzyme production, the variables with confidence levels greater than 95% were considered as significant. CMC was most significant for cellulase production.

Cellulases produced from bacterial and fungal strains have traditionally been estimated by significant CMC as reported by several authors (Ghos 1987; Kim 2008).

#### Effect analysis by Pareto chart

**Figure 2:**
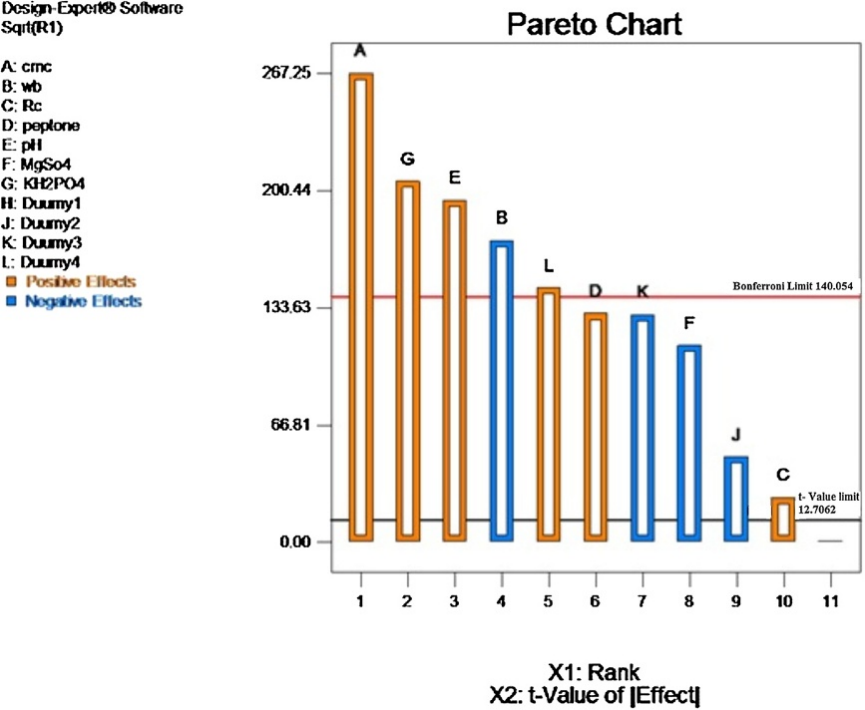
**Pareto chart showing the effect of media components on cellulase activity.** Developed from the data analysis in the Plackett-Burman experimental run, this chart was used to determine the significant factors affecting cellulase yield.

The purpose of the Pareto chart (Figure 2) is to highlight the significance of CMC among all (typically large) set of factors. Table 3 shows the positive and negative effects of media components on cellulase activity. As it can be seen that B -wheat bran and F- MgSO_4_ (Blue color) have negative effect in comparison to other components, both of these have been eliminated in optimization procedure of CCD.

**Table 3:** **Statistical analysis of Plackett-Burman design showing coefficient values,** ***t*** **-value and** ***P*** **-value for each variable**

Variable	Coefficient	t-value	P value
Intercept	1.08	12.7062	0.0049*
CMC	0.24	12.7062	0.0024*
WB	−0.15	4.30265	0.0037
RC	0.025	3.18245	0.0249
Peptone	0.12	2.77645	0.0049*
pH	0.17	2.57058	0.0033*
MgSO_4_	−0.099	2.44	0.0057
KH_2_PO_4_	0.18	2.36462	0.0031*

*Significant factors.

From the Pareto analysis it could be possible that only CMC had a significant impact at 95% as compared to other parameters at activity level. The parameter values were also compared with enzyme (AVICEL) and the conditions were validated here in this work as evinced by Goldbeck et al. (2013).

### Optimization of medium components by response surface methodology

At the end of screening experiments by Plackett-Burman design, 4 factors were found to play a significant role in cellulase production. Table 4 summarizes the response of various run experiments performed for ANOVA for the statistical design of experiment. To test the fit of the model equation, the regression-based determination coefficient (R^2^) was evaluated.

**Table 4:** **CCD experimental design**

	Factor 1	Factor 2	Factor 3	Factor 4	Response (Activity(IU/ml))	
Run	A:CMC(g/l)	B:KH2PO4(g/l)	C:pH	D:Peptone(g/l)	Predicted	Actual
1	12	3	7	2.5	2.9	1.86
2	10	2	6	1.5	2.1	1.74
3	10	2	6	3.5	1.8	1.95
4	12	3	3	2.5	0.8	0.58
5	12	3	5	2.5	5.14	4.97
6	12	3	5	2.5	5.08	4.97
7	10	4	6	1.5	0.4	0.21
8	10	2	4	1.5	0.6	1.12
9	10	4	6	3.5	0.7	0.5
10	10	2	4	3.5	0.2	0.95
11	12	3	5	0.5	2.6	1.96
12	12	3	5	2.5	4.84	4.97
13	10	4	4	3.5	1.4	0.85
14	12	3	5	2.5	4.78	4.97
15	14	4	4	3.5	0.8	1.47
16	8	3	5	2.5	2.6	2.09
17	14	2	4	1.5	0.5	0.89
18	12	5	5	2.5	0.2	0.21
19	16	3	5	2.5	3.1	2.38
20	12	3	5	2.5	4.8	4.97
21	12	3	5	2.5	5.12	4.97
22	14	2	4	3.5	0.1	0.1
23	14	4	6	1.5	2	1.37
24	12	3	5	4.5	2.1	1.51
25	12	1	5	2.5	1.8	0.55
26	14	4	4	1.5	2.3	2.18
27	14	2	6	3.5	0.7	1.01
28	14	2	6	1.5	0.9	1.43
29	10	4	4	1.5	0.9	0.94
30	14	4	6	3.5	1.7	1.03

#### ANOVA for CCD (Central Composite Design)

Analysis of variance for cellulase activity of *T. reesei* (Table 4) revealed that CMC and other factor variation had a significant influence (p ≤ 0.001) based on hydrolyzing capabilities of the strain. The F-value of 8.54 implies that the model is significant. A^2,^ B^2^, C^2^, D^2^ are significant model terms to test the fit of the model equation as revealed from its F value. The results of the second-order response surface model are fitting in the form of ANOVA (Table 5).

**Table 5:** **Analysis of variance table [Partial sum of squares - type III]**

Source	Sum of squares	Df	Mean square	F value	P-value
Model	72.28	14	5.16	8.54	<0.0001
A-CMC	0.15	1	0.15	0.25	0.6251
B-KH2PO4	0.0004167	1	0.0004167	0.0006894	0.9794
C-pH	2.47	1	2.47	4.09	0.0614
D-Peptone	0.45	1	0.45	0.75	0.3999
AB	2.18	1	2.18	3.6	0.0772
AC	0.005625	1	0.005625	0.0093	0.9244
AD	0.39	1	0.39	0.65	0.434
BC	1.38	1	1.38	2.28	0.1515
BD	0.005625	1	0.005625	0.0093	0.9244
CD	0.11	1	0.11	0.17	0.6818
A^2^	12.86	1	12.86	21.28	0.0003
B^2^	36.10	1	36.10	59.74	<0.0001
C^2^	23.97	1	23.97	39.66	<0.0001
D^2^	17.99	1	17.99	29.76	<0..0001
Residual	9.07	15	0.60		
Lack of fit	8.92	10	0.89	20.63	0.0007
Pure error	0.14	5	0.029		
Cor total	*81.34*	29			

The final equation in terms of coded factor was given as


Statically optimized medium used for the production of other microbial cellulose using *Gluconacetobacter persimmonis* was carried out by Hegde et al. (2013). To the extent, it could be an alternative approach for media optimization in the case of *Trichoderma spp*. Hegde et al. (2013) and Rajendran et al. (2007) have followed PBD for screening of the medium constituents (slightly different) and a CCD for optimization of significant factors. Here authors have employed different factors as per the demand of equations were estimated as significant factors from PBD. The synchronized properties of medium components and their optimum regions are reported in this manuscript.

#### Response surfaces

**Figure 3:**
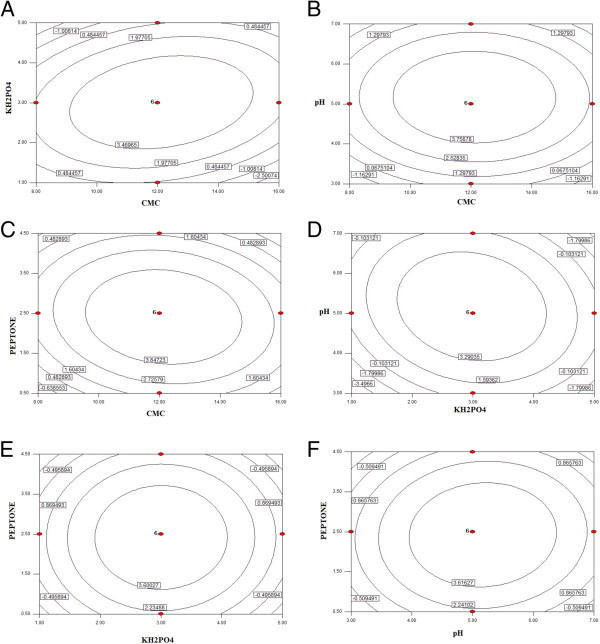
**Contour plot for cellulase production showing the interactive effects of medium components. (A)** Interaction plot of CMC and KH_2_PO_4_. **(B)** Interaction plot of CMC and pH. **(C)** Interaction plot of CMC and Peptone. **(D)** Interaction plot of pH and KH_2_PO_4_. **(E)** Interaction plot of Peptone and KH_2_PO_4._ **(F)** Interaction plot of Peptone and pH. These contour plots are actual 2D projections of response plots which are developed in the methodology used in the present study. They give the idea of interactions between variables affecting the yield.

The interaction effects and optimal levels of the variables were determined by plotting the three-dimensional (3D) response surface curves. Response surfaces were made for analyzing the response pattern at any point. The response surface curve in Figure 3A represented the interaction between CMC (A) and KH_2_PO_4_ (B) which exhibited that the maximum enzyme activity was obtained in middle range (12 g/l) while enzyme activity decreased in lower and higher range of CMC probably due to increase in viscosity of the medium on increase in concentration of CMC In addition, lower and higher levels of KH_2_PO_4_ resulted in lower cellulase activity. The higher and lower concentration of KH_2_PO_4_ may have affected pH of the medium and hence affected the cellulase activity. The shape of the response surface curves showed strong positive interaction between these tested variables. Figure 3B represented response surface plot of two independent variables that is CMC (A) and pH (C). It was inferred that maximum cellulase synthesis took place when concentration of CMC was kept at their middle level. The pH optima for enzyme production from earlier sources were in the range of 4.5–5.7. Figure 3C represented response surface plot between CMC (A) and peptone (D). It can be seen that maximum cellulase activity was obtained when values of carbon and nitrogen sources were kept at their respective intermediate level. Interaction between variables can also be predicted by visualizing their respective contour plot. The effect of varying concentration of KH_2_PO_4_ (B) and pH (C) on enzymatic activity at constant CMC and peptone is shown in Figure 3D. The typical plots were dome shaped. Figure 3E shows the interactive surface of KH_2_PO_4_ (B) and peptone (D). The nitrogen source (peptone) in concentration range of 2 g/l to 2.5 g/l showed elevation in the curvature of the response plot while KH_2_PO_4_ optimum range 2.5 g/l to 3.5 g/l. concentration of peptone below 2 g/l showed green color and depression in the curve of plot. Figure 3F represents the response surface of peptone and pH, other variables i.e. CMC and KH_2_PO_4_ were kept constant. The optimum pH range (5.0-5.5) was much narrowed in comparison to the response plot discussed before.

Contour plots (Figure 3) indicated the general shapes of the response surface. However, the contour plots are two-dimensional, whereas the current RSM study had four variables, so two variables must be set at an arbitrary value and the response surface plotted with the other two variables. Elliptical nature of contour indicated the significant interaction between variables while circular contour showed insignificant interaction between variables. The resulting Response surface was the function of the value chosen for the variable held constant and may differ considerably, depending on what value is selected.

Application of RSM in the optimization of analytical methods is presented in this investigation. The multivariate statistical technique of RSM and steps for its application are adapted as described by rsearchers (Bezerra et al. 2008; Saravanan et al. 2012).

### Validation experiment

Experiments in duplication were performed to validate the developed optimized medium. The optimized medium recorded a higher enzymatic activity of CMCase as compared to other basal media. Overall results indicated that a sharp fold (1.63X) increase occurred after optimization (Table 6). These results confirmed the validity of the optimized medium. 1.85 and 1.88 fold increase was obtained in BGL and FPA respectively. Overall an average of 1.85 fold increase was obtained (Table 6).

**Table 6:** **Optimization summary of enzymes**

	Activity(IU/ml)		
	CMCase	BGL	FPA
Unoptimized	3.142	0.66	1.38
Optimized	5.140	1.22	2.60
Fold increase	1.6	1.85	1.88

### Molecular characterization

#### SDS-PAGE analysis

**Figure 4:**
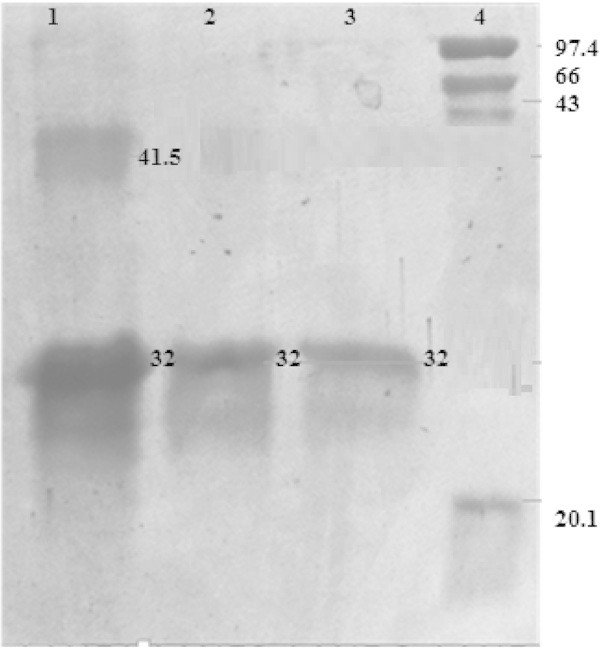
**SDS PAGE analysis of** ***Trichoderma reesei*** **cellulase composition Lane1; crude** ***cellulase*** **, Lane 2 purified** ***cellulase*** **, Lane 3 purified** ***cellulase*** **lane 4 Standard marker (GeNei).**

The crude dialysed enzymes samples obtained through dialysis membrane were resolved on a 5% stacking and 15% running gel. *Trichoderma reesie-*cellulase complex (endo-glucanase, exo-glucanse and BGL) was found to be homogenous scattered view of crude monomeric protein, as evidenced by the multiple bands corresponding to the designated size of SDS page (Figure 4). Analysis of different molecular weight of putative cellulase monomers are very much based on the available reports pertaining to the genetic information of many species*.* The molecular of protein determined by SDS –PAGE was very close to molecular weight of cellulase of *T. viride* 31.2 KDa (Sultana et al. 1997) and endoglucanase molecular weight(23-42 KDa) as described (Abdullah et al. 2005). The cellulase was found to be much active in pH range of 4.8-5.5 while temperature range varied from 50°C to 70°C.

#### Zymography

**Figure 5:**
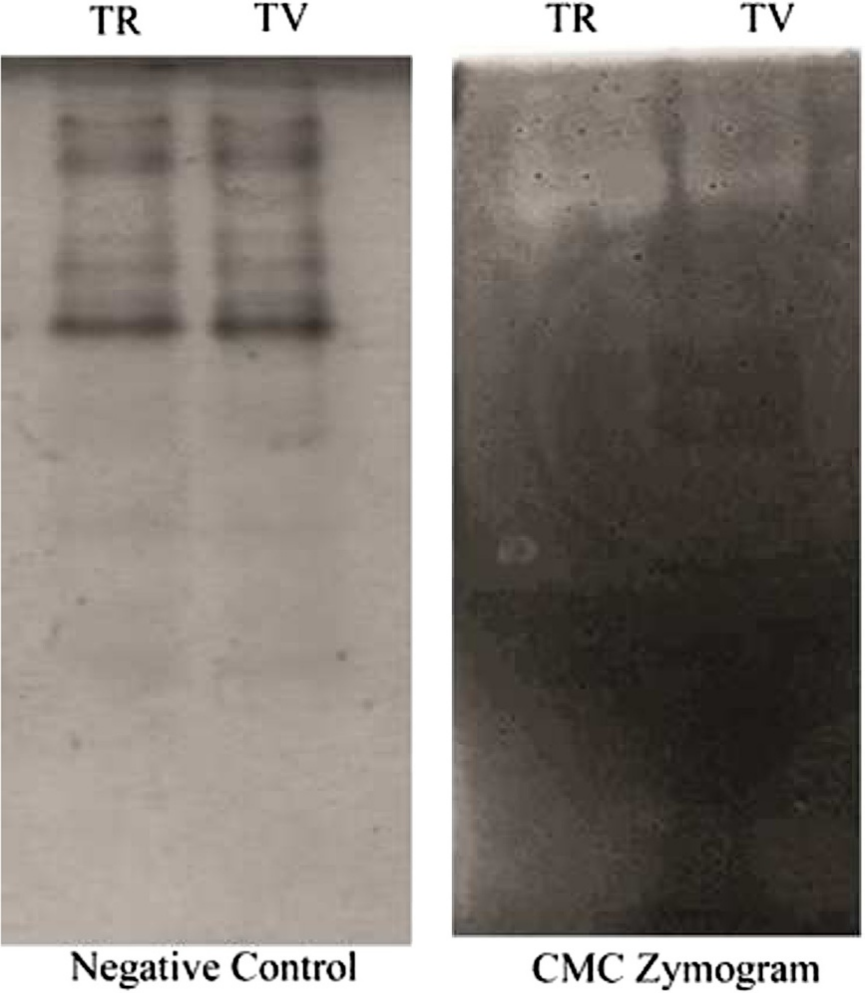
**Cellulase zymogram of** ***Trichoderma reesei*** **(TR) and** ***Trichoderma viride*** **(TV) on CMC and negative control.**

Figure 5 clearly shows that, it is a complex system containing different components. *T. reesei* (Figure 5) has shown that high enzymatic activity on CMC when compared with *T. viride.*

#### Circular dichroism

**Figure 6:**
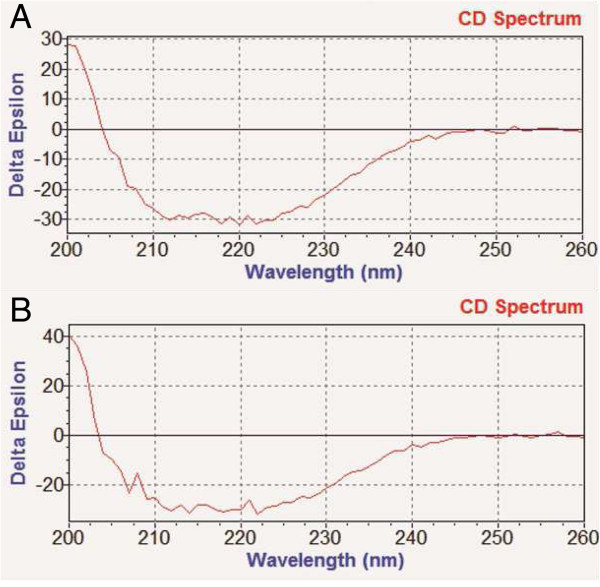
**CD curves of cellulase samples from (A)** ***Trichoderma reesei,*** **(B)** ***Trichoderma viride.***

Circular dichroism is a method that would account for the secondary structure content in a polypeptide based on the presence of alpha helix, beta sheet and random coil (Keideiling 1996). We probed the secondary structure of Cellulase by far-UV CD (Table 7). Two deep minima near 208 nm and 222 nm characteristic of α helical structure in aqueous solution are due to π-π* & n-π* transitions. β-sheet is having negative at 218 nm (π-π*). From Figure 6, predominantly α-helical (more than 60%) was detected for Cellulase at pH 7.4. At pH 7.4, far-UV CD spectra of Cellulase clearly reflect characteristics of (α) type of structure.

**Table 7:** **Secondary structure composition of cellulase determined from Far-UV CD spectra in pH 7.4 at 25°C**

(a)	
Secondary structure	Amount
Helix	100%
Antiparallel	0
Parallel	0
Beta Turn	1.3%
Random Coil	0.2%
**(b)**	
**Secondary structure**	**Amount**
Helix	100%
Antiparallel	0
Parallel	0
Beta Turn	1.2%
Random Coil	0.2%

(a) Cellulase from (a) *Trichoderma reesei,* (b) *Trichoderma viride.*

## Conclusion

Significant data obtained in this investigation could be stated that: the predicted value achieved by RSM that is 4.96 IU/ml, is in close agreement with observed value that is 5.14 IU/ml. After the employment of laboratory bench scaling and optimization methodology approximately 2 fold increase in activity was observed. Molecular weight as determined by SDS page is almost identical to molecular weight of other cellulose and endoglucanase well characterized by Sultana et al. and Abdullah et al. (Sultana et. al. 1997; Abdullah et al. 2005). *T. reesei* shows highest enzymatic activity when compared with *T. viride* on CMC substrate. Cellulase secondary structure composition is very similar in all analysed species. Further studies on the performance of these proteins and their mutants on the growth of plant will uncover the mechanism and potential of these proteins.

## Abbreviations

RSM: Response surface methodology
IU/ml: International unit/Millilitre
CMC: Carboxymethlyl cellulose
BGL: β glucosidase
EG: Endoglucanase
CR: Congo red
RC: Root crush
CCD: Central composite design
FPAse Assay: Filter paper assay
CD: Circular dichroism.
